# Filamentous Aggregates of Tau Proteins Fulfil Standard Amyloid Criteria Provided by the Fuzzy Oil Drop (FOD) Model

**DOI:** 10.3390/ijms19102910

**Published:** 2018-09-25

**Authors:** Dawid Dułak, Małgorzata Gadzała, Mateusz Banach, Magdalena Ptak, Zdzisław Wiśniowski, Leszek Konieczny, Irena Roterman

**Affiliations:** 1 ABB Business Services Sp. z o.o. ul. Żegańska 1, 04-713 Warszawa, Poland; dawid.dulak@gmail.com; 2 ACK—Cyfronet AGH, Nawojki 11, 30-950 Kraków, Poland; m.k.gadzala@gmail.com; 3 Department of Bioinformatics and Telemedicine, Medical College, Jagiellonian University, Łazarza 16, 31-530 Kraków, Poland; mateusz.banach@uj.edu.pl (M.B.); magdalena.ptak@uj.edu.pl (M.P.); mywisnio@cyf-kr.edu.pl (Z.W.); 4 Faculty of Physics, Astronomy and Applied Computer Science, Jagiellonian University, Łojasiewicza 11, 30-348 Kraków, Poland; 5 Chair of Medical Biochemistry, Medical College, Jagiellonian University, Kopernika 7, 31-034 Kraków, Poland; mbkoniec@cyf-kr.edu.pl

**Keywords:** tau amyloid, Alzheimer’s disease, tauopathy

## Abstract

Abnormal filamentous aggregates that are formed by tangled tau protein turn out to be classic amyloid fibrils, meeting all the criteria defined under the fuzzy oil drop model in the context of amyloid characterization. The model recognizes amyloids as linear structures where local hydrophobicity minima and maxima propagate in an alternating manner along the fibril’s long axis. This distribution of hydrophobicity differs greatly from the classic monocentric hydrophobic core observed in globular proteins. Rather than becoming a globule, the amyloid instead forms a ribbonlike (or cylindrical) structure.

## 1. Introduction

The origin of amyloid transformation has attracted scientific attention for more than 35 years—at least since being acknowledged as the cause of various neurodegenerative disorders [1]. The coexistence and mutual relations between Aβ amyloids and tau tangles, resulting in the damage and destruction of synapses, is believed to provoke behavioral changes that are associated with cognitive impairment [2,3]. The appearance of amyloid fibrils is the consequence of plasticity of proteins, which can adopt different conformational states [4]. The proteins of high content of intrinsically disordered structural forms seem to be the candidates ready for partially folded state which may transform to disordered aggregates with low packing [5,6].

Emergence of fibrillary structures is also thought of as the result of involvement of intrinsically disordered proteins, especially at early phases of the folding process [7].

Reaching the form of highly packed structuralised aggregates that are based mainly on β-structural forms opens the possibility for the unlimited elongation of highly packed ordered amyloid form [8]. The presence of Beta-structural form (cross Beta) allows for the propagation due to the possible H-bonds system to be organised on both sites.

The specificity of tau amyloidosis, as evidenced by abnormal phosphorylation, results not only in disorganization of microtubulin, but also in the appearance of intracellular tau filaments, referred to as neurofibrillary tangles [9]. Tauopathies are defined as clinically, morphologically, and biochemically heterogeneous neurodegenerative diseases that are characterized by the deposition of abnormal tau protein in the brain [10].

Tau amyloid fibrils are regarded as peculiar due to the existence of two distinct superfibrillary forms: straight filament and paired helical filament [11]. Two individual protofilaments may form different structures depending on their mutual arrangement in the dimer. The authors of [11] refer to the conformation of the protofilament as “C-shaped”. The dimer (superfibril), when analyzed under cryo-electron microscopy (cryo-EM) imaging [12], resembles two arched C-shaped structures that are bound back to back. In one form, the structure is symmetrical (with the same residues in both unit molecules involved in complexation), while the other form lacks such symmetry (in this case, the central fragment of one chain makes contact with a fragment which is somewhat closer to the N terminus of the adjacent chain).

According to the analysis shown in [11], the dimerization of protofilaments occurs by way of hydrogen bonds forming between adjacent fragments. In its native form, tau in complex with a microtubule adopts a conformation referred to as “natively unfolded”. This conformation is highly resistant to spikes in temperature and acidity [13].

The presented here analysis focuses on microtubule-associated tau neurofibrillary tangle protein, paired helical filaments (consisting of two individual fibrils), individual protofibrils, as well as individual chains.

Identification and characterization of amyloid structures, as shown in this work, bases on comparative analysis of the structure of globular and fibrillary proteins. The proposed fibrillarization model for globular structures is also discussed in [14,15]. At the core of the fuzzy oil drop (FOD) model lies the concept of applying a three-dimensional (3D) Gaussian form to express the idealized distribution of hydrophobicity in a globular protein. Such distribution has a distinct peak at the geometric center of the globule and then falls off along with distance from the center, reaching almost zero at the surface (i.e., at a distance of 3σ from the center). We have identified proteins whose structure closely corresponds to this theoretical distribution [16]. Any local deviations are usually associated with the protein’s intended function: local excess of hydrophobicity, if occurring on the surface, usually marks a complexation interface, while the hydrophobicity deficiencies tend to correspond to binding cavities, which are capable of housing ligands (or substrates, in the case of enzymes) [17,18]. The universality and ubiquity of hydrophobic cores is attested to by a study of a large number of proteins with varying secondary and supersecondary structural characteristics [19]. A specific type of discordance vs. the idealized (monocentric) distribution of hydrophobicity is observed in the case of proteins which contain so-called solenoid fragments, including some antifreeze proteins [20]. Such fragments deviate from a centralized core in favor of a distribution comprising alternating bands of high and low hydrophobicity, propagating along solenoids long axis [20]. However, in addition to solenoids, biologically active proteins also include fragments whose purpose is to restrict unchecked propagation of such linear sequences (thereby preventing dimerization or polymerization), as well as to ensure solubility. The special “caps” are identified in such proteins [20]. Structures that strongly deviate from the monocentric distribution and lack suitable “caps” are prone to amyloid filaments [14,15]. In the context of the FOD model, such structures can be likened to ribbonlike micelles, capable of unrestricted propagation [21].

A thorough description of the FOD model can be found in [22].

In the FOD model, the emergent structure of the hydrophobic core is though to result from interactions between the protein and the aqueous solvent, which directs hydrophobic residues towards the center of the globule and favors exposure of hydrophilic residues on the surface. As already indicated, local discordances are often associated with the presence of external factors. The fact that amyloid forms do not require mutations to emerge suggests that misfolding is not caused by factors internal to the polypeptide itself. One shall mention the mutation-related amyloidosis [23], however the prion-based amyloid transformation does not require the presence of mutation, as it is the discussed case.

Shaking is known to promote amyloidogenesis—and it can hardly be called a chemical factor. Many factors—including chemical ones—were identified to support the amyloidosis transformation [24]. However other factors than environmental (shaking in particular) are not the object of our analysis. One shall also take into account that the folding as well the misfolding processes take place in macromolecular crowding conditions, however the immanent presence of water makes the water environment of high importance [25].

Perhaps shaking disrupts the structure of the solvent in such a way as to prevent it from guiding “natural” conformational changes within the protein chain. Alternatively, shaking is notable for aerating the solvent. The resulting increase in the area of the liquid/gas interface may produce structural changes within the solvent itself.

In addition to analysis of the tau amyloid, as listed in Protein Data Bank [26], this work proposes an in silico experiment, which involves determining alternative structures that the tau amyloid sequence may attain (using specialized protein folding software, such as Robetta [27,28] and I-Tasser [29,30]), and performing folding simulations based on the FOD model. It turns out that the sequence is indeed capable of producing a globular form with a single, monocentric hydrophobic core. Subjecting globular structures to FOD characterization enables us to track changes that result in amyloidogenesis. The work focuses on three distinct structures: (1) the superfibril (seeking the causes behind its structural variability); (2) the protofibril (identifying the characteristic properties of amyloid structures); and (3) a single chain participating in the fibril. Our research is based on observations rooted in the FOD model, specifically, the linear propagation of hydrophobicity in amyloids (which prevents a shared hydrophobic core from forming). As discussed in [14,15], the presence of alternating bands of high and low hydrophobicity can be regarded as one of the principal indicators of amyloid transformation.

## 2. Results

### 2.1. Abbreviations Used

FOD—Fuzzy Oil Drop model
RD—Relative Distance—The divergence entropy introduced by Kullback and Leibler (described in Methods) used to express the distance between two compared profiles is of entropy category thus it requires an introduction of reference distribution. This is why the distance between T-O (T theoretical—idealized distribution and O-observed distribution) measured by divergence entropy is compared with the O-R (O-observed, R-uniform distribution deprived on any form of hydrophobicity concentration), also measured by divergence entropy. The parameter expressing the relative distance O|T versus (O|T + O|R) measures the closeness of O distribution versus T distribution in respect to O versus R distribution. The RD parameter becomes polypeptide chain length independent. It makes possible comparison of different proteins.
RD (T-O-R)—RD parameter calculated for two reference distributions T-theoretical and R-uniform
RD (T-O-H)—RD parameter calculated in respect to reference distribution called H-distribution based on intrinsic hydrophobicity of amino acids present in particular polypeptide chain fragment
HvT—correlation coefficient expressing the relation between H-intrinsic hydrophobicity of amino acids versus the T-theoretical (expected) hydrophobicity for the idealized status of the residue
HvO—correlation coefficient expressing relation between H-intrinsic hydrophobicity of amino acid versus is status as observed in particular protein
TvO—correlation coefficient expressing relation between T-idealized hydrophobicity and O-observed in protein under consideration
phf-tau—paired helical filament-tau
phf-tauO—paired helical filament-tau—as it is available in 5O3O phf-tau in symmetrical form of superfibril
phf-tauT—paired helical filament-tau—as it is available in 5O3T phf-tau in asymmetrical form of superfibril
phf-tauL—paired helical filament-tau—as it is available in 5O3L phf-tau in form of superfibril similar to phf-tauO
IT-#—identification of the model constructed using I-Tasser program with number 1–5 since 5 models were constructed using this program
ROB-#—identification of the model constructed using Robetta program with number 1–5 since 5 models were constructed using this program
FOD-#—identification of the model constructed using FOD model with number 1–5 since 5 models were constructed using this program
Tau (267–312)—fragment of tau peptide—protein under PDB ID 2MZ7
Tpp—tau phosphothreonine peptide—protein under PDB ID 1I8H
Tau (306–311A)—fragment of tau to identify the structure available in PDB as 2ON9
Tau (306–310)—fragment of tau to identify the structure available in PDB as 3Q9G
Tau (306–311B)—fragment of tau to identify the structure available in PDB as 3OVL
Tau (305–311)—fragment of tau to identify the structure available in PDB as 4E0M
Tau (623–628)—fragment of tau to identify the structure available in PDB as 4NP8
Tau (306–311C)—fragment of tau to identify the structure available in PDB as 5K7N
F-actin—actin, alpha skeletal muscle as available in PDB as 3J8I
PDB—Protein Data Bank
CASP—Critical Assessment Protein Structure Prediction
BLAST—Basic Local Alignment Search Tool
PSI-BLAST—Position-Specific Iterated BLAST
MSA—Multiple Sequence Alignment

### 2.2. Superfibril

This analysis concerns the amyloid form that is listed in PDB as 5O3O, 5O3L, and 5O3T (pronase-treated paired helical filament in Alzheimer’s disease brain neurofibrillary tangle protein, paired helical filament-tau, phf-tau, *Homo sapiens*). Fragment: residues 623–695 of tau protein (306–378 according to PDB numbering) Chains A, C, E, G, I, along with their counterparts (B, D, F, H, J) make up the proto-fibrils [11]. In order to characterize individual chains in the context of the superfibril and proto-fibrils, we have singled out chains E and F. These two chains are located in the central part of the fibril and can be regarded as representative of an arbitrarily long structure. This selection also minimizes edge effects caused by the finite width of the complex.

#### Properties of Superfibrils and Interfaces—What Is the Source of Different Isoforms of Tau Filaments?

Table 1 presents the status of tau amyloid structures in terms of RD values, revealing large discordances between T and O profiles in both models (T-O-R and T-O-H). This means that the distribution of hydrophobicity does not involve a central hydrophobic core. Further analysis will reveal that the amyloids are dominated by a pattern that consists of alternating bands of high and low hydrophobicity. High values of RD further indicate that the folding process is driven by the intrinsic properties of each residue rather than by a global force field—this is also typical for amyloids [14,15]. Regarding the hydrophobicity profile correlation coefficients, HvT and TvO lag behind HvO. This is also due to the absence of a central hydrophobic core, which is replaced by linear propagation of narrow “bands”. In further sections we will specifically describe locations that exhibit these properties.

**Figure 1:**
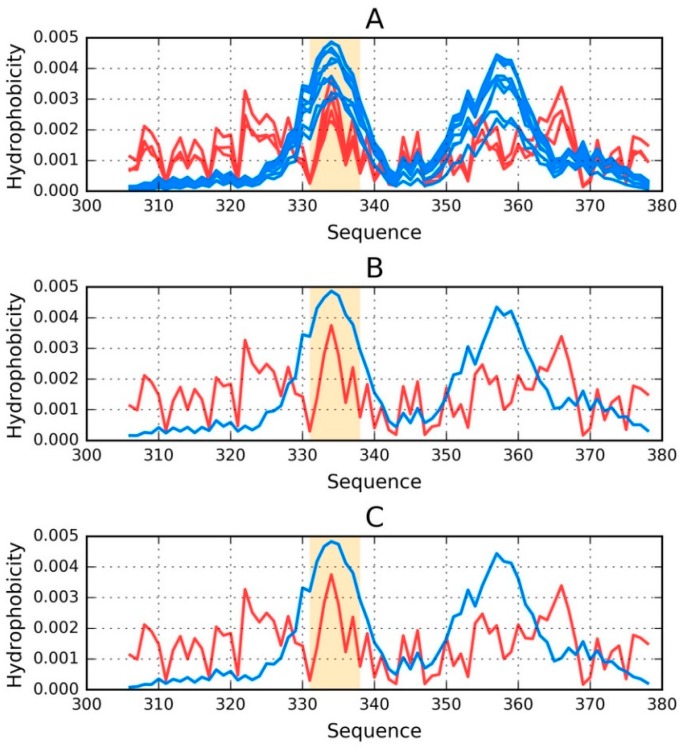
Theoretical (T—blue) and observed (O—red) distributions of hydrophobicity in: (**A**) the phf-tauO superfibril; (**B**,**C**) chains E and F as components of the phf-tauO superfibril. Orange highlighting marks residues which comprise the inter-fibril interface area (331–338).

Visual comparison of T and O (Figure 1) highlights the major differences between these distributions. It should be noted that the chart consists of many overlapping profiles, which means that the distribution of local minima and maxima is replicated in each adjacent chain, resulting in a set of narrow bands, as suggested above.

The FOD model may also be used to predict the properties of shared hydrophobic cores in protein complexes [31]. In order to properly characterize a given complex, it is important to assess the status of its interface. With regard to proto-fibrils, the distribution that was observed in phf-tauT differs from those exhibited by the remaining structures. However the difference is limited only to the structure of interface, which is discussed in this paper. In phf-tauO and phf-tauL the status is similar and it suggests that the superfibril emerges as a result of factors consistent with the FOD model, i.e., under the influence of the aqueous solvent. This interpretation is supported by the high values of all correlation coefficients. We may conclude that the interface is shaped by all factors which determine the structure of the complex itself, with major involvement of water.

The picture changes, however, when dealing with phf-tauT. Its high value of RD (T-O-R), coupled with negative values of HvT and TvO coefficients and a high value of the HvO coefficient, suggest that, in this case, the solvent does not play a significant role in complexation.

It should be noted that the status of the interface is computed by taking into account all interface residues in the entire fibril (following protein-protein contact distance criteria of PDBsum [32]). When all three correlation coefficients adopt strongly positive values, we may assume that the structure of the interface represents a compromise between all three hydrophobicity profiles (observed, intrinsic and theoretical). In contrast, negative values of HvT and TvO are understood to mean that the interface folds “in spite of” the FOD model and in consequence in spite of environmental effects that act upon the protofibril complex.

The characterization concerns chains E and F, which are located centrally and therefore representative of an unrestricted fibril. The calculated values are typical for amyloid forms, and include high values of RD and HvO, along with very low (sometimes even negative) values of HvT and TvO. In phf-tauT, both individual chains, as well as the interface fragment, are shaped by intrinsic hydrophobicity rather than by the external environment, which would favor the formation of a monocentric hydrophobic core.

As the status of individual chains (in the context of the superfibril) is largely similar in all structures, we will limit their presentation to Phf-tau in form, as observed in phf-tauO and phf-tauT (Table 2).

When analyzing individual chains as components of the superfibril, we arrive at similar RD values. In contrast, when the same chains are analyzed as components of individual proto-fibrils (phf-tauT), their values differ due to differences in the orientation of each proto-fibril. Negative values of correlation coefficients for HvT and TvO with a high value of correlation coefficient for HvO relation suggest that phf-tauT is a typical amyloid form.

Figure 1 provides a graphical representation of the superfibril and both chains (E and F) treated as components of the superfibril.

The status of phf-tauO superfibril is visualized in Figure 1A, which shows that the theoretical distribution involves two local maxima, along with hydrophilic fragments that are exposed on the surface. Neither maximum is evident in the observed distribution, however O includes other local maxima, located in areas where low hydrophobicity is expected. It should be noted that each of these local maxima (as well as minima) represents an entire band stretching along the fibril’s long axis. The overlap is due to the repeating pattern that is present in each individual chain, with only the outlying chains exhibiting slightly lower hydrophobicity. On the other hand, the differences between the theoretical distributions are readily apparent since this distribution predicts that hydrophobicity should decrease along with distance from the center. The degree of discordance between T and O can be analyzed by comparing theoretical charts with the observed distributions for chains E and F (which are centrally located and therefore representative for the entire fibril—see Figure 1B,C).

The interface fragment appears to be consistent with the FOD model. Given the central location of the interface, a hydrophobicity peak is expected and—to a certain extent—present in the actual complex. Comparing O with T reveals that two outlying residues exhibit relatively low hydrophobicity, while the central section corresponds to a major spike. Consequently, we rate the interface fragment as being accordant with the model.

A characteristic feature of amyloids is the presence of numerous local maxima in areas where low hydrophobicity (and vice versa) is predicted by the theoretical model. However, it is important to remember that, unlike globular proteins (which may also exhibit this phenomenon), the complexed chains form here bands which stretch along the entire long axis of the fibril. These observations are confirmed by analysis of T and O for chains E and F (treated as components of the superfibril). The discordance between T and O distribution in most of proteins is of local character.

### 2.3. Properties of Proto-Fibrils

Table 3 presents the hydrophobicity parameters for each proto-fibril. In this case, each proto-fibril is treated as a distinct structural unit. This means that a separate Gaussian is constructed for each proto-fibril (in the preceding section, a shared Gaussian form was computed for the entire superfibril). Results are indicative of an amyloid form: high values of RD and HvO along with very low (even negative) values of HvT and TvO. The correlation coefficients reveal that the structure is dominated by the intrinsic properties of its component residues—an observation that is supported by the observed high values of RD in both models (T-O-R and T-O-H). Thus, the observed distribution is more closely aligned with R (or H) rather than T.

The status of chains E and F, treated as components of their respective proto-fibrils, confirms that they adopt amyloid-like forms, although this effect is less pronounced than in the case of the superfibril (lower values of T-O-H RD and HvO—see Table 3).

**Figure 2:**
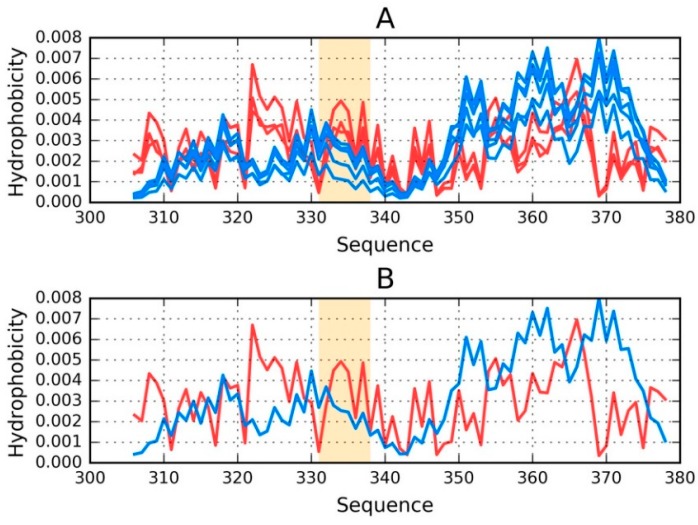
Theoretical (T—blue) and observed (O—red) distributions: (**A**) as calculated for the proto-fibril (chains A, C, E, G and I) listed under phf-tauO; (**B**) for the E chain treated as a component of the proto-fibril. Orange highlighting marks residues which comprise the inter-fibril interface (331–338).

Figure 2 provides a visual representation of these results, showing T and O distributions for one proto-fibril (chains A, C, E, G and I) and the status of the E chain within this structural unit.

An asymmetrical distribution of local maxima is observed in the proto-fibril as a result of significant displacement of the system’s central point as compared to the superfibril. Numerous local maxima are present in areas where low hydrophobicity is expected. The involvement of a local maximum in the interface fragment indicates that complexation of protofibrils is generated as the effect of the influence of environment (according to FOD model).

### 2.4. Properties of Individual Chains Treated as Distinct Structural Units

Our analysis also covers individual chains that are treated as distinct structural units, with a separate Gaussian being plotted for each chain (under the assumption that each chain folds in separation from other chains). To determine the causes of the discordance between the observed and theoretical distributions, we have singled out fragments for which this discordance is particularly evident. Note that we are not dealing with isolated deviations—in many areas, both distributions strongly oppose each other, indicating that the chain does not produce a globule and is likely insoluble due to the lack of a polar surface.

**Figure 3:**
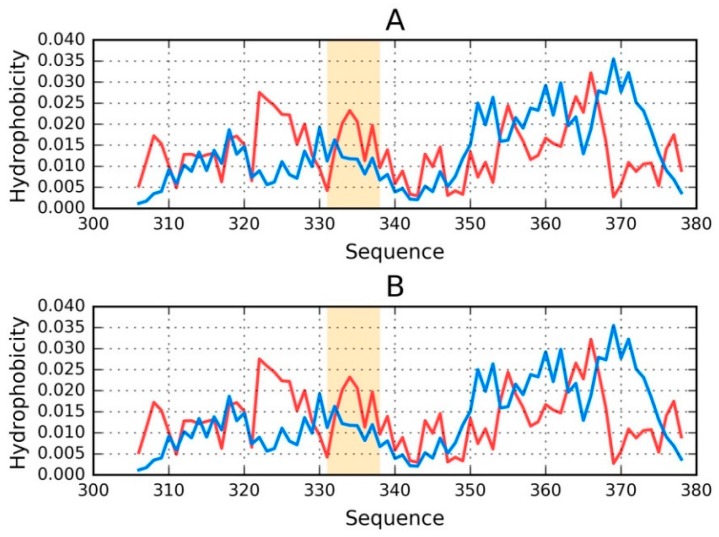
Theoretical (T—blue) and observed (O—red) distributions for phf-tauO: (**A**) chain E treated as a distinct structural unit; and (**B**) chain F treated as a distinct structural unit. Orange highlighting marks residues which comprise the inter-fibril interface (331–338).

Figure 3 illustrates the status of chains E and F treated as individual structural units.

It is clear that even when analyzed as distinct units, the discussed chains still diverge from the theoretical distribution of hydrophobicity (Figure 3). No C-terminal maxima (predicted by T) are present in the observed distributions.

Regarding phf-tauL, both of the distributions are similar to those calculated for phf-tauO, with only the interface being somewhat different. The observed distribution, while discordant, does not resemble an amyloid (which would appear as a sinusoidal pattern consisting of similar local maxima).

**Figure 4:**
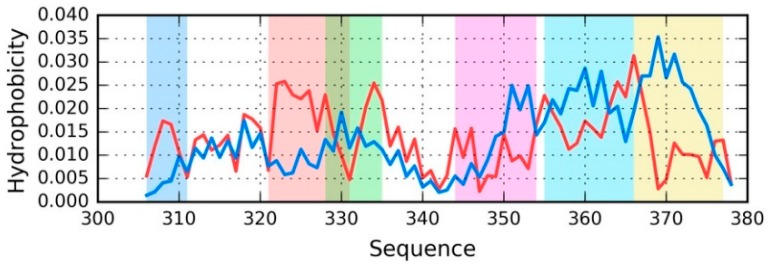
Theoretical (T—blue) and observed (O—red) distribution for the chain E from phf-tauO (the distribution is similar in all structural units). Fragments where O significantly deviates from T are highlighted by different colors (see Table 4) that match the colors of three-dimensional (3D) presentations on Figure 5.

Taking into account the discussed distributions, it is easy to pinpoint fragments where O deviates from T (see Figure 4).

Figure 4 provides a sample set of distributions (T and O) for a single chain—E from phf-tauO. As already noted, the chains differ in detail, while the overall pattern remains largely identical, regardless of the structural unit in question (superfibril, protofibril, or individual chain). The highlighted fragments have been singled out on the basis of visual inspection, supplemented with correlation coefficients computed for successive five-residue segments. Fragments for which HvT and TvO are negative while HvO assumes that a large value will be subjected to further analysis.

Table 4 summarizes the results obtained for all structural units in phf-tauO.

As shown in Table 4, the status of selected fragments is quite similar, regardless of the structural unit in question—in all cases, these fragments are strongly discordant vs. the theoretical distribution.

### 2.5. Comparative Analysis Involving Theoretical Models

As previously noted, we have carried out an in silico experiment that consisted of predicting the conformation of a tau protein whose sequence matches the discussed amyloids. Our analysis concerned the entire molecule as well as fragments that are highlighted in Figure 4.

**Figure 5:**
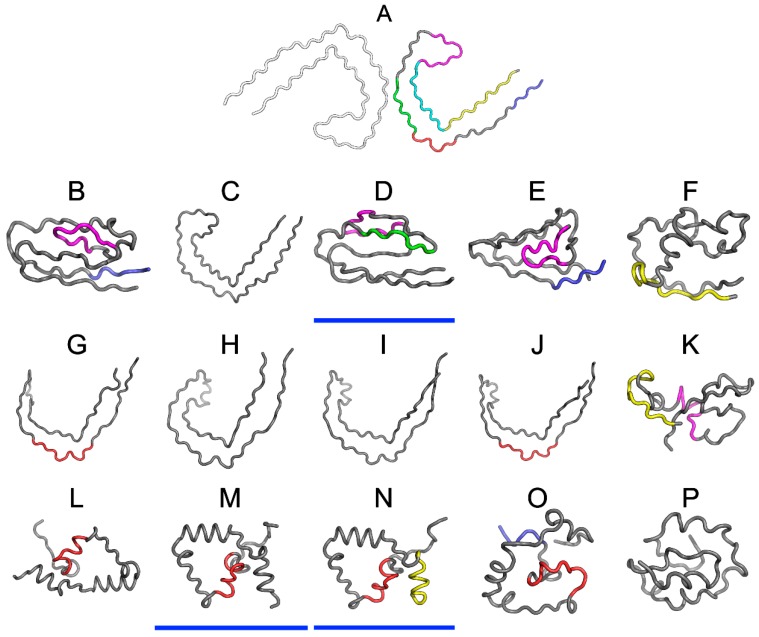
3D presentation of structures obtained using I-Tasser upper row (**B**–**F**), Robetta central row (**G**–**K**) and the fuzzy oil drop (FOD) model bottom row (**L**–**P**). The top structure (**A**) is the structure phf-tauO as appears in PDB database. The colors used correspond to highlights on Figure 4. Models whose status is consistent with FOD predictions (RD < 0.5) have been additionally underlined in blue (**D**,**M**,**N**).

Figure 5 presents 3D models of tau polypeptides obtained using software packaged described in the Materials and Methods section. Visual inspection reveals the possible emergence of globular forms: I-Tasser produces four such structures (out of five input cases), while Robetta produces one (out of five). The tendency of the FOD model to produce globular forms should come as no surprise given the model’s propensity to direct hydrophobic residues towards the center of the molecule (due to interactions with the aqueous solvent).

One of the models that was produced by I-Tasser appears to involve a hydrophobic core (in the sense of the FOD model—cf. underlined structures in Figure 5). None of the models produced by Robetta satisfies this criterion. Regarding the FOD model, despite its natural tendency to generate hydrophobic cores, only two among 500 structures analyzed in the course of the study contain a hydrophobic core (i.e., satisfy the RD < 0.5 condition).

Analysis of numerical values that are listed in Table 5, along with visual inspection of 3D forms reveals that some of these fragments adopt structures consistent with the Gaussian distribution. Of particular note is the fragment marked in red on Figure 5 (residues 50–61, numbered 356–357 according to PDB), which does not conform to theoretical predictions in any model. In interpreting this fact, we may refer to the dominant role of the fragment whose sequence does not adapt to the centralized distribution of hydrophobicity. We can speculate that this fragment (GSLDNITHVPGG) is therefore the most amyloidogenic sequence in the set under consideration. This suggestion is supported by the data shown in Table 5, particularly RD values that are either highest or second highest in the entire set.

### 2.6. Other Fragments of Tau Proteins

In order for our comparative analysis to be as comprehensive as possible, we also include tau proteins (or fragments of tau amyloids) that PDB lists as being capable of adopting non-amyloid conformations. The Tpp (1I8H) represents the 541–553 fragment of the previously described tau protein, in complex with a microtubule—specifically, ww domain complexed with human tau phosphothreonine peptide microtubule-associated protein tau. In this complex, the A chain comprises residues 541–553 (so-called phf-tau), while chain B represents the ww domain (6–44) [33].

The Tpp sequence does not fully match phf-tau (and the others), however we have included it in our analysis due to functional similarities.

**Figure 6:**
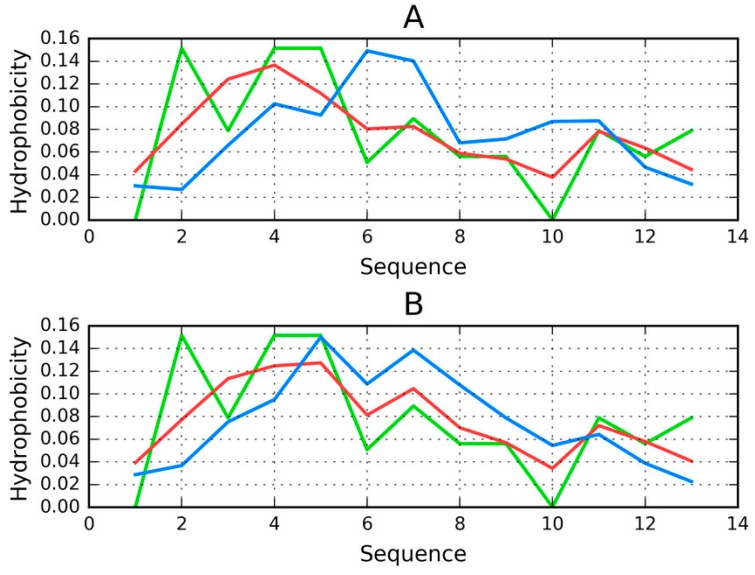
Distribution of hydrophobicity in chain A in the Tpp in complex (T—blue, O—red, H—green): (**A**) when treated as a distinct structural unit; (**B**) when treated as a component of the complex. Good alignment between O, T, and H can be observed in the latter case.

Results shown in Table 6 and on Figure 6 reveal that the Tpp complex does not conform to the FOD model. The status of chain A (tau), when analyzed on its own, is also discordant. On the other hand, the same chain conforms to the model when analyzed as part of the complex. This means that chain B creates suitable conditions for chain A to produce a shared hydrophobic core that is consistent with the 3D Gaussian.

Considering the individual fragments of Tpp, it turns out that the fragment 27–29 of chain B is the most discordant, and that eliminating this fragment from calculations lowers the RD value of the complex. This may indicate the conformational alignment between chain B and chain A (given that the presence of chain A disrupts the distribution of hydrophobicity in chain B).

Analysis of our results indicates the need of a “chaperone”, which chain A requires to reach together a conformation consistent with the FOD model.

Tau (267–312) is another protein related to the discussed tau structure. Its sequence matches the short fragment at 306–312 in tau as it is present in phf-tauO. According to [34] this fragment (267–312) of tau protein is bound to microtubules.

**Figure 7:**
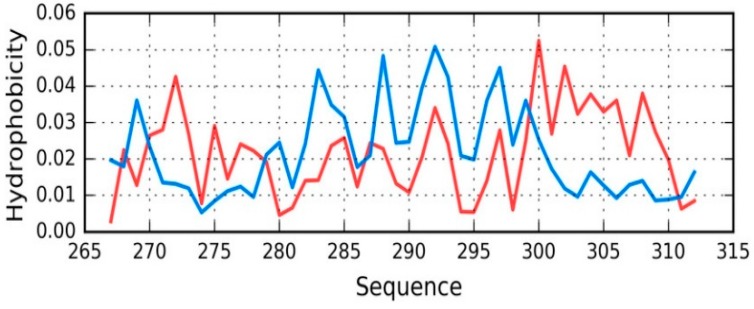
T (blue) and O (red) hydrophobicity distributions in Tau (267–312) (2MZ7), revealing overall strong discordance.

The status of the tau chain in tau (267–312) reveals strong discordance with regard to the theoretical model (see Figure 7), with RD = 0.680 (T-O-R) and 0.527 (T-O-H). Correlation coefficients are −0.049, −0.042, and 0.673 for HvT, TvO, and HvO, respectively. These values indicate that the structure of the chain is dominated by the conformational tendencies of individual residues rather than by the external hydrophobic force field. Interaction with microtubules is likely to be the driving force behind conformational adaptation. The structure of the entire complex is not known, however information regarding the interaction of individual residues with the microtubule might explain the discordance that was observed throughout the chain. Only the helical fragment at 295–299 appears accordant with the FOD model (with RD values and correlation coefficients of 0.264, 0.170, 0.876, 0.921, and 0.965, respectively, showing good alignment between the theoretical and observed distribution).

### 2.7. Peptides

To complete our study of tau-derived structures that are listed in PDB we also need to consider peptides capable of amyloid transformation. The possible mechanism driving this process, discussed in [14,15], remains applicable in the case under consideration.

Peptides that match the tau protein sequence are mostly related to the fragment at 306–311—the short N-terminal fragment of the tau protein present in phf-tauO, phf-tauL, and phf-tauT. A short peptide which does not produce a globular form should not, in principle, be analyzed using the FOD model. Nevertheless, for the sake of completeness, we will present RD (T-O-R) values calculated for such peptides—see Table 7.

The values shown in Table 7 only reveal the type of hydrophobicity distribution, with no assessment of the hydrophobic core structure. Low values indicate that hydrophobic residues are located in the central part of the chain, surrounded by N- and C-terminal hydrophilic residues. As shown, the short VQIVYK fragment, despite including an outlying Val residue, is a good match for the centralized hydrophobic core structure. When additional neighboring residues (adjacent to the 306–311 fragment) are included in analysis, the value of RD increases significantly.

**Figure 8:**
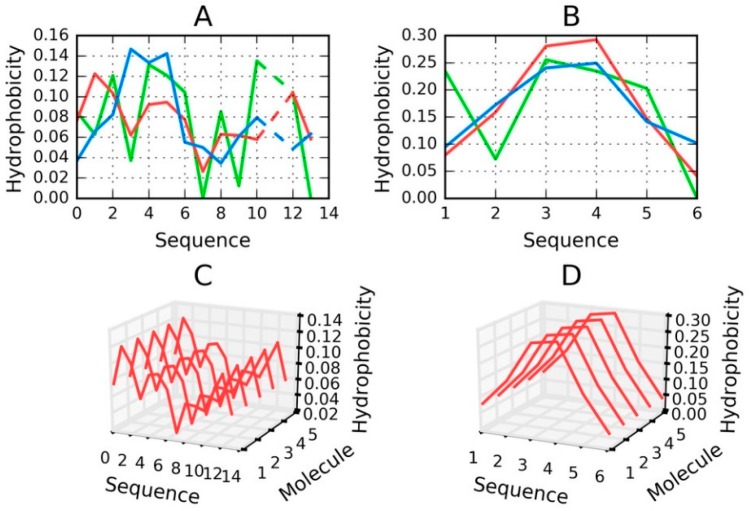
H (green), T (blue) and O (red) distributions for selected peptides: (**A**) Tau (305–311), (**B**) Tau (306–311B); (**C**) for (**A**) and (**D**) for (**B**)—pseudo-3D view, presenting the observed hydrophobicity of a theorized structure of fibrillary tangles formed by linear propagation of the corresponding peptides. Dashed lines on (**A**) represent deleted residue number 11 Tau (305–311) according to data available in PDB.

Peptides that are identified as capable of amyloidogenesis appear to adopt amyloid-like conformations themselves. As discussed in [14,15], the distribution of hydrophobicity in a peptide may—regardless of its accordance with the theoretical distribution—give rise to amyloid formation, as long as the environment favors linear complexation of additional peptides, with alternating bands of high and low hydrophobicity emerging along the axis of the fibril. This is visualized in Figure 8, which compares two fringe cases (in terms of RD values).

Figure 8 evidences the appearance of structures that are characterized by linear propagation of hydrophobicity peaks/troughs, which is a precondition of amyloid formation. As highlighted by to-date observations and interpretations, the environment must “support” the creation of such forms. It is thought that under natural conditions the structure of water does not favor the formation of amyloid fibrils.

The value of RD computed for a short peptide implies how many local maxima are present. A low value indicates that hydrophobicity is concentrated in the central part of the peptide (e.g., Tau (306–311B)), while a high value suggests the presence of numerous local maxima (e.g., Tau (305–311)).

### 2.8. Is It Possible to Differentiate between the Amyloid Fibril and the Fibrillary Structure Present in the Microfilament?

The defining property of amyloids is their fibrillary nature. This phenomenon, however, is not restricted to amyloids. Many biologically active fibrillary proteins exist, often serving as biological scaffolds—this includes polymer microfilaments, such as F-actin (filamentous actin). An example structure of this protein is listed in PDB under ID 3J8I [35].

Analysis of protein 3J8I reveals an alternative approach to a fibrillary structural formation that relies on different mechanisms than those, which drive amyloidogenesis. The PDB structure comprises five monomeric units arranged into a linear complex. Each monomer is a single-domain chain, 375 aa in length, with varying secondary folds: three beta sheets (11 beta strands in total) and 17 helices. The monomers are spatially arranged in a shape of a helix, forming an elongated fibril. Our analysis focuses on the F chain, which is placed in the center of this fibril. We believe that this chain best represents any inner subunit of a long fibril, with adjacent neighbors on either side.

In analyzing the F chain we apply a twofold approach: first, we treat the chain as an independent structure (constructing a 3D Gaussian capsule calculated specifically for that chain), and subsequently we analyze it as part of the complex (with a broader Gaussian encapsulating the entire complex). The former approach enables us to determine the status of the chain itself, while the latter provides clues regarding its role in the formation of a fibril.

The same observations that we relied on when an analyzing the tau amyloid (high values of RD in both configurations; negative HvT and TvO correlation coefficients and strongly positive HvO) will be sought in our study of F-actin to determine whether the conditions that give rise to amyloid fibrils also apply in the presented case.

The above mentioned set of parameters shows that T not only deviates from O, but can, in some respects, be viewed as its polar opposite. This is taken as evidence that the given conformation is driven by intrinsic hydrophobicity of individual residues.

**Figure 9:**
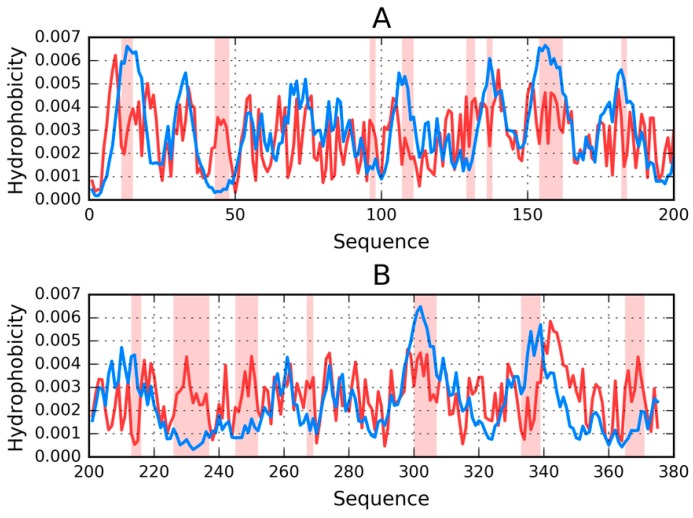
T (blue) and O (red) profiles for the F chain from F-actin (PDB ID 3J8I), divided into two parts for visibility: 1–200 (**A**) and 201–375 (**B**). Highlighted positions mark residues that cause discordance between those distributions (on the basis of visual inspection). The remainder of the chain is regarded as accordant with the theoretical distribution. It likely contributes to the protein’s structural stability—under the assumption that a well-ordered hydrophobic core and the presence of disulfide bonds both play a role in stabilizing tertiary conformations.

Table 8 presents FOD parameters that describe F-actin (as listed in PDB under ID 3J8I), which were derived from T and O distributions plotted in Figure 9. It appears that the entire complex, as well as both versions of the F chain, deviate from the theoretical distribution, with no monocentric hydrophobic core being observed in either case. Note that Table 8 only lists the status of selected fragments—those, whose amyloid-like conformation may be important in light of the current discussion regarding identification of amyloid forms.

Results show that local amyloid-like properties may be attributed (in varying degrees) to the beta sheet. Such localized amyloid-like folds can indeed be found in many biologically active proteins (e.g., antifreeze proteins that contain solenoid fragments) [36]. Likewise, the beta sheet found in the lysozyme may also be regarded as amyloid-like [37]. (Note that this particular beta sheet plays an important role given its proximity to the active site—it even contributes one of the catalytic residues of the lysozyme). It seems that the presence of a similar structure in actin is not a unique phenomenon, especially given the structure of its immediate neighborhood. As it turns out, local amyloid-like folds in biologically proteins are typically bracketed by “stop” signals (or “caps”), which prevent unchecked linear propagation. They do so by ensuring that the structure, as a whole, conforms to the theoretical distribution of hydrophobicity and mediating entropically advantageous contact with water. This is highlighted in Table 8 with “stop” annotations.

**Figure 10:**
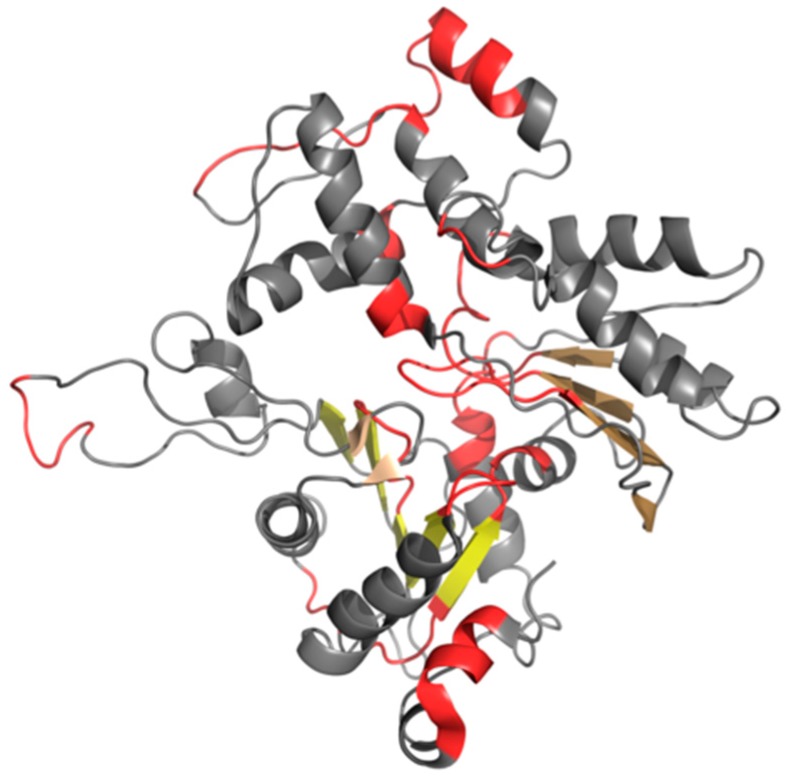
3D presentation of F chain from F-actin (PDB ID 3J8I). Beta sheets are displayed in different shades of yellow. Red fragments distinguish residues that cause discordance between T and O distributions. These fragments correspond to highlighted parts of hydrophobicity profiles presented in Figure 9.

In the scope of our analysis we also computed FOD correlation coefficients for successive fragments of the input chain while using a 5 aa moving frame. This reveals the exact placement of residues which exhibit amyloid-like characteristics. Eliminating such residues lowers the value of RD (although not below 0.5—see the “No neg CC” annotations in Table 8). Visual inspection of both profiles (theoretical—T and observed—O) reveals residues that contribute to the discordance (these are highlighted in Figure 9 and marked in Figure 10, which presents the protein’s 3D structure). It is worth noting that these residues together comprise only 10% of the chain. Eliminating visually inspected residues brings RD down below 0.5, which means that the remainder of the chain conforms to the monocentric distribution of hydrophobicity.

The amyloid-like beta sheet-1 is characterized by the linear propagation of alternating bands that differ in terms of hydrophobicity. This effect manifests itself as a strong discordance between T and O profiles, where—in some cases—the observed distribution appears to be a polar opposite of the theoretical distribution.

**Figure 11:**
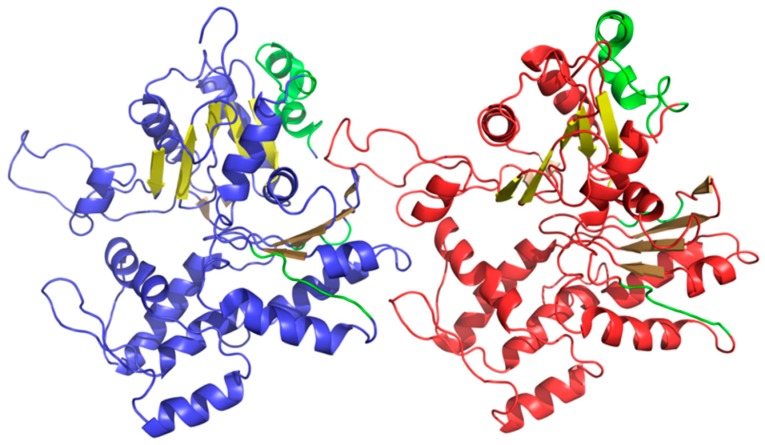
3D presentation of F chain (blue) and H chain (red) from F-actin (PDB ID 3J8I). Beta sheets are displayed in different shades of yellow (same three shades in each chain). “Stop” fragments are shown in green.

Linear propagation can be observed by studying the status of successive fragments that comprise the beta sheet. It is therefore interesting to speculate about the participation of such beta sheets in formation of a complex with a clearly fibrillary nature. The sheets in question are dispersed and do not form a continuous band of alternating hydrophobicity. Consequently, they cannot be regarded as a structural scaffold for the complex. What is more, the beta sheets that are contributed by different chains are not in contact (as shown in Figure 11). The presence of “stop” fragments, also shown in Figure 11, which arrest linear propagation, suggests that amyloid-like conditions are intended to remain local and not dominate the structure. Similar “caps” can be found in many other proteins, which include amyloid-like fragments [38] and prevent the unrestricted elongation of such structures.

In summarizing our comparative analysis of fibrillary structures, it should be noted that these structures owe their existence to different mechanisms. An amyloid emerges as a consequence of linear propagation of alternating bands of high and low hydrophobicity, whereas globular proteins form complexes via nonbinding interactions (including salt bridges and hydrogen bonds). In the latter case, even when amyloid-like fragments can be found in the proteins’ structures, they are dispersed and protected by “stoppers”, which prevent them from interacting with one another to form complexes.

In effect, we can state that the structure referred to as a “fibril” might be produced in various ways. Linear propagation of hydrophobicity bands is the prerequisite of amyloid formation (as well as a useful criterion for identifying amyloids), whereas other fibrillary structures (such as F-actin) are formed through nonbinding interactions (including salt bridges and hydrogen bonds). Thus, even though the end result (elongated fibril) is similar, the underlying mechanisms differ. Against this background we propose that the criteria listed in this book differentiate amyloids and enable their identification. We also show that the presence of beta folds is not required (e.g., as evidenced by the tau amyloid). Instead, amyloids may form whenever the folding process is driven by the intrinsic properties of individual residues, as confirmed by the parameters that are studied in this work.

## 3. Discussion

The comparative analysis of proteins associated with amyloid tau confirms the previously stated hypothesis concerning the structural properties of the amyloid. According to this hypothesis, the amyloid is characterized by the presence of alternating bands of variable hydrophobicity. It seems that linear propagation—which can be regarded as contrary to the emergence of a centralized hydrophobic core (as seen in globular proteins)—is a characteristic property of amyloids. A similar phenomenon can be observed in Aβ amyloids [39,40]. The network of hydrogen bonds that is discussed in numerous studies [11,41] favors this type of conformation and is thought to be associated with the linear properties of beta folds. In the tau amyloid, however, β-strands play a much smaller role than in other known amyloids. This suggests that while hydrogen bonds are important, their role is not necessarily linked to β-structures.

Hydrophobicity is capable of binding together proximate charged residues, however, electrostatic interactions should, in principle, prevent such clustering. Under such conditions only hydrophobic forces can result in the observed arrangement. Thus, a conformation that is driven by intrinsic hydrophobicity (and does not generate a central hydrophobic core) may be regarded as both the cause and the mechanism of amyloid transformation.

The FOD model recognizes several possible forms for the tau superfibril. This diversity is likely caused by interactions between the solvent and the emerging amyloid. We suggest that, while phf-tauO and phf-tauL emerge as the effect of the influence of surrounding water, in phf-tauT, the structure is driven by the specific band-like arrangement of hydrophobicity in the amyloid itself.

The tau protein, whose task is to mediate interaction with microtubules, must align itself to the complexed object. When the protein is subjected to folding on its own, in an independent manner, it may adopt a globular conformation and remain soluble. An open question is why the same protein undergoes complexation in a form which does not resemble a globule. As shown, a chain that is sequentially identical to the amyloid fragment of the tau chain cannot produce a globular structure. In this context, microtubules may be viewed as a “chaperone”, which ensures that the protein adopts its intended conformation, required for biological activity.

Conclusions that are related to the process of amyloidogenesis and the role of the FOD model in explaining this process, all point to the need for further research into the properties of the aqueous solvent. While we possess good knowledge of the properties of ice, the corresponding “normal” (or physiological) condition of liquid water is poorly understood—for example, we are still unsure of why the density of water peaks at 4 degrees C. This may explain the recent uptake in investigations that aim to explain such phenomena [41,42,43,44,45,46]. We believe that these studies may also cast a new light on the process of amyloidogenesis, which—in all likelihood—is associated with the (heretofore unknown) influence of the force field exerted by the surrounding water. This field should be modeled as a continuum rather than (as is common practice in modern molecular dynamics packages) as a collection of distinct molecules. The FOD model provides a good baseline for such research.

The analysis allows for distinguishing of critical short sequences especially resistant to adopt the conformation accordant with the expected uni-centric hydrophobic distribution. This phenomenon is also observed in other amyloids, especially Aβ(1–42) amyloid [39].

## 4. Materials and Methods

### 4.1. Data

The analysis concerns tau protein amyloids listed in PDB as capable of forming highly ordered superfibrils. In addition, we also consider selected fragments of the tau protein, including short peptides. Table 9 gives the full list of structures subjected to analysis.

Table 8 includes tau superfibrils (phf-tauL, phf-tauO, phf-tauT), smaller structural units (including individual chains—tau (267–312)), as well as complexes with other proteins (Tpp). We also consider individual peptides that are widely characterized as capable of forming amyloid structures.

All of the above structures are subjected to FOD characterization in the context of the superfibril, the protofibril and the individual chain. Our analysis further extends to peptides whose composition is similar or identical to PDB sequences. The status of such molecules is determined by computing their RD coefficients. It should be noted that seeking proper hydrophobic cores in very short peptides (<15 aa) makes little sense—such peptides are characterized while using FOD criteria only in order to provide a coherent platform for comparative studies. The FOD model provides useful information regarding the relationship between each residue’s intrinsic hydrophobicity and its placement in a fully folded chain.

### 4.2. Folding of Peptides—Components of Amyloid Structures

Peptide sequences which form parts of the tau amyloid (e.g., 306–378, as listed under phf-tauO) have been subjected to folding simulations while using Robetta [26,28] and I-Tasser [29,30], as well as to simulations based on the FOD model [53]. This operation can be regarded as an in silico experiment whose aim is to provide alternatives to structures generated by specialized 3D structure prediction software. Our goal is to identify theoretical opportunities for alternative folds (unlike those listed under phf-tauO and similar entries). The globular forms that are generated by the FOD model may provide clues regarding the discordance between the theoretical distribution of hydrophobicity and the actual location of hydrophobicity maxima/minima. A ranking list of the resulting structures may be composed in order to identify factors that increase similarities between the theorized conformation and the corresponding amyloid form.

Robetta is a software package that is aimed at the modeling and analysis of protein structures [27,28]. It is a strong performer in successive editions of the CASP challenge, which focuses on predicting the 3D conformations of input residue sequences [54]. Robetta works in the following manner: the user is asked to input a sequence of amino acids comprising a given protein chain. This sequence is then subdivided into fragments (called domains) while using the “Ginzu” hierarchical scanning algorithm. The algorithm recognises fragments homologous to sequences for which the preferred secondary conformation has been established on the basis of experimental studies. Such homologous areas are detected by (in the order of accuracy) BLAST, PSI-BLAST [55], FFAS03 [56], and 3D-Jury [57] taking as input the sequences produced in the preceding step. The identified domains are modeled by applying a comparative modeling protocol, while all other chain fragments are treated as linkers (if they consist of fewer than 50 residues) or are assigned to structural families as defined in the Pfam-A database [58] using HMMER [59]. Fragments and sequences that have not been recognized as putative domains are analyzed via MSA of the full-length target derived from a PSI-BLAST search against the NCBI non-redundant (NR) protein sequence database [60]. Putative domains identified through Pfam-A and MSA are modeled using de novo structure prediction. Finally, following assembly, side chains are modeled by applying Monte Carlo algorithms [61]. The description is based on [60].

I-Tasser (Iterative Threading ASSEmbly Refinement) is a software package which can predict the structure of a protein given its sequence. In this application, prediction bases on querying PDB for templates using the multiple threading approach. I-Tasser is a strong contender in CASP challenges, topping the ranking in editions 7 through 12 [62,63,64].

The user submits a sequence of amino acids, which is then compared (by LOMETS [65]) to template proteins with similar structural characteristics. An optimal template is then selected and overlapping fragments are assembled into an output model while using replica-exchange Monte Carlo simulations [66], while differing fragments are modeled ab initio. If LOMETS is unable to identify a suitable template, the entire structure is subjected to ab initio modeling. The next step involves a search for low energy states (using SPICKER [67]) in the resulting chain via clustering simulation decoys. This is followed by the reassembly of the template protein starting with SPICER cluster centroid, however this time the simulation is guided by spatial constraints that are provided by TM-align on the basis of LOMETS templates and PDB data. The purpose of the second iteration is to remove steric clashes as well as to refine the global topology of the cluster centroids. The decoys generated in the second simulations are clustered and the lowest energy structures are selected [68]. The final step involves the construction of a detailed model from the available structures via optimization of the hydrogen bond network using REMO. Further information can be found in [68].

Robetta computations were carried out while using the publicly available service [27]. I-Tasser computations were carried out using [68]. FOD model computations were carried out using PL-Grid platform on “Cyfronet” Computer Center AGH Krakow infrastructure [69], a detailed description of which can be found in [70].

The FOD model involves two intermediate folding stages: the early-stage intermediate [71,72,73,74] and the late-stage intermediate [72,73,74]. The initial step, which is meant to generate a starting structure for further optimization, is omitted since the conformation listed under 5O3O is taken as the starting structure (treated as early-stage in this case). This chain is then immersed in an aqueous solvent, whose effects are modeled using a 3D Gaussian (as an external force field). In line with the FOD model, hydrophobic residues tend to congregate at the center of the protein body while hydrophilic residues are exposed on its surface. The process produces a prominent hydrophobic core that is encapsulated by a hydrophilic “shell” (with near-zero values of hydrophobicity on the surface). Optimization of hydrophobic interactions and optimization of nonbinding internal interactions is carried out in an alternating fashion, with each step being repeated several times.

Nonbonding interactions are optimized using Gromacs 4.6.5 software suite (Groningen, The Netherlands) [75], available on the PL-Grid infrastructure at ACK Cyfronet AGH Kraków [69]. FOD-based optimization aims to minimize differences between the idealized (3D Gauss function) and observed distribution of hydrophobicity in the target protein. The workflow interleaves both procedures in order to converge on the final conformation.

Folding simulations that rely on the FOD model are relevant since they acknowledge the effects exerted by the aqueous solvent, and treat them as a global phenomenon (i.e., external force field producing a molecule-wide hydrophobic core).

### 4.3. Comparative Analysis

All tertiary conformations that were produced by the modeling algorithms, as well as structures that are listed in PDB, were analyzed with regard to the status of their hydrophobic cores, which is described by the RD (relative distance) coefficient. Comparing RD values brings the information about the degree of disorder in respect to ideal distribution. In consequence, the approach to amyloid form can be assessed. RD expresses the degree of order present in the protein’s hydrophobic core and indirectly indicates whether the protein is globular or not. Generating a globular structure with a prominent hydrophobic core (hydrophobicity peaking at the center of the molecule and decreasing along with distance from the center, becoming very low on the surface) suggests that the given amyloid peptide may, under certain circumstances, adopt a globular conformation. The ranking of protein structures, sorted in the order of decreasing globularity, reveals changes which cause proteins to forfeit their centralized hydrophobic cores and that may—in extreme cases—produce amyloid forms.

The RD coefficient can be computed for two independent cases: T-O-R and T-O-H. The former case expressing the relative distance between the observed distribution (O) and two boundary distributions: theoretical (T) which is given by the 3D Gaussian, and uniform (R, random) where each residue is ascribed a hydrophobicity value of 1/N (N being the number of residues in the input chain). R-distribution represents the case of uniform (absence of any local hydrophobicity concentration) distribution, which is the opposite one versus the centralized distribution. In the latter case the uniform distribution is replaced by a distribution corresponding to the intrinsic hydrophobicity of each residue in the input chain (H). Comparing both values reveals factors that guide the folding process (this is particularly true in the T-O-H case). A high value of RD (T-O-H) indicates that folding is dominated by the intrinsic properties of each residue with no regard to cooperative generation of a shared hydrophobic core. When this type of distribution is repeated in successive fragment of the polypeptide, the result is a linear sequence of alternating bands of high and low hydrophobicity. This, in turn, enables the unrestricted elongation of the fibril. An interpretation of this phenomenon (referred to as “ladders”) can also be found in [76].

A comparative assessment of T-O-R and T-O-H coefficients in fibrillary/amyloid structures as well as the structures that are produced by various folding algorithms may enable us to identify the “seeds” of linear propagation. FOD criteria have previously been used to assess the distribution of hydrophobicity in structures published by the CASP project [77].

It is hard to compare the interpretation based on FOD with other methods due to the fact that the hydrophobic interaction is underestimated in the discussion concerning amyloid transformation. However, some aspects of intrinsically disordered proteins that were extensively investigated [78] remain in agreement with the results of the analysis of these proteins in respect to the FOD model [79]. The development of techniques as cryo-electron microscopy [80] as well as solid state NMR [81] makes the availability of amyloid structures possible. Structuralization of water is recently in focus of attention, especially the ordering of water on surface [82] what remains in close relation to our model 

## Figures and Tables

**Table 1:** Relative Distance (RD) values and correlation coefficients computed for the superfibril, proto-fibrils and individual chains treated as components of the superfibril.

Phf-tau	RD		Correlation Coefficient		
	T-O-R	T-O-H	HvT	TvO	HvO
phf-tauO	0.745	0.687	0.012	0.259	0.675
phf-tauL	0.731	0.669	0.013	0.296	0.646
phf-tauT	0.724	0.641	0.008	0.301	0.716
Inter-fibril interface					
phf-tauO	0.401	0.550	0.666	0.754	0.950
phf-tauL	0.368	0.587	0.696	0.772	0.929
phf-tauT	0.527	0.386	−0.164	−0.003	0.850
Chains in superfibril					
Phf-tauO					
Chain E	0.741	0.700	0.011	0.237	0.731
Chain F	0.761	0.722	0.010	0.224	0.731
Chains E and F	0.752	0.711	0.013	0.230	0.731
Phf-tauL					
Chain E	0.728	0.675	0.017	0.274	0.696
Chain F	0.747	0.698	0.012	0.265	0.696
Chains E and F	0.741	0.690	0.014	0.268	0.606
Phf-tauT					
Chain E	0.732	0.666	0.034	0.400	0.769
Chain F	0.728	0.666	−0.018	0.136	0.785
Chain E and F	0.730	0.666	0.008	0.273	0.777

**Table 2:** Status of individual chains treated as components of the superfibril. The presentation of phf-tauL is omitted since due to its similarity to phf-tauO.

Chain	RD				Correlation Coefficient					
	T-O-R		T-O-H		HvT		TvO		HvO	
	Phf-tauO	Phf-tauT	Phf-tauO	Phf-tauT	Phf-tauO	Phf-tauT	Phf-tauO	Phf-tauT	Phf-tauO	Phf-tauT
A	0.780	0.801	0.696	0.681	0.010	0.041	0.154	0.441	0.669	0.710
B	0.787	0.739	0.711	0.600	0.031	−0.022	0.303	0.115	0.636	0.723
C	0.756	0.766	0.715	0.705	0.014	0.038	0.202	0.394	0.731	0.772
D	0.779	0.728	0.741	0.665	0.022	−0.020	0.248	0.114	0.730	0.788
E	0.741	0.732	0.700	0.666	0.016	0.034	0.237	0.400	0.731	0.769
F	0.761	0.728	0.722	0.666	0.010	−0.010	0.224	0.136	0.731	0.785
G	0.733	0.700	0.690	0.628	0.016	0.028	0.265	0.394	0.731	0.770
H	0.745	0.733	0.704	0.670	0.003	−0.017	0.192	0.157	0.732	0.786
I	0.727	0.660	0.613	0.522	0.015	0.022	0.343	0.405	0.652	0.686
J	0.750	0.738	0.634	0.618	−0.019	−0.014	0.137	0.192	0.691	0.707

**Table 3:** Status of individual chains treated as components of their respective proto-fibrils. The table lists only values obtained for the proto-fibril (chains A, C, E, G and I) (Differences with regard to the other proto-fibril are negligible.

PDB ID	RD		Correlation Coefficient		
	T-O-R	T-O-H	HvT	TvO	HvO
Phf-tauO	0.661	0.607	−0.012	0.089	0.772
Chain E	0.679	0.430	−0.027	0.095	0.548
Chain F	0.679	0.439	−0.027	0.095	0.548
Phf-tauL	0.664	0.595	−0.022	0.082	0.767
Chain E	0.674	0.410	−0.039	0.091	0.545
Chain F	0.674	0.410	−0.039	0.091	0.545
Phf-tauT	0.673	0.602	−0.017	0.118	0.773
Chain E	0.683	0.415	−0.033	0.098	0.550
Chain F	0.683	0.415	−0.033	0.096	0.551

**Table 4:** RD values in both models (T-O-R and T-O-H), along with HvT, TvO, and HvO correlation coefficients for chains E and F analyzed as part of the superfibril, as part of a protofibril and on their own. Figure 4 illustrates the division of the chain into individual fragments.

Phf-tauO					
**Chain E—Superfibril**					
**Fragment**	**RD**		**Correlation Coefficient**		
	**T-O-R**	**T-O-H**	**HvT**	**TvO**	**HvO**
1–6	0.521	0.405	0.207	0.270	0.855
16–26	0.820	0.771	−0.126	−0.491	0.874
23–30	0.496	0.495	−0.350	0.435	0.580
39–49	0.575	0.544	0.040	0.147	0.971
50–61	0.851	0.802	−0.106	−0.782	0.446
61–72	0.563	0.491	−0.157	−0.024	0.700
CHAIN	0.741	0.700	0.016	0.237	0.731
**Chain F—Superfibril**					
**Fragment**	**RD**		**Correlation Coefficient**		
	**T-O-R**	**T-O-H**	**HvT**	**TvO**	**HvO**
1–6	0.627	0.512	0.038	0.117	0.875
16–26	0.822	0.773	−0.129	−0.495	0.875
23–30	0.496	0.495	−0.318	0.465	0.580
39–49	0.563	0.533	0.054	0.153	0.971
50–61	0.861	0.814	−0.112	−0.720	0.444
61–72	0.583	0.512	−0.153	0.006	0.699
CHAIN	0.761	0.722	0.010	0.224	0.731
**Chain E—Protofibril**					
**Fragment**	**RD**		**Correlation Coefficient**		
	**T-O-R**	**T-O-H**	**HvT**	**TvO**	**HvO**
1–6	0.668	0.553	−0.006	0.064	0.875
16–26	0.615	0.557	0.288	−0.141	0.875
23–30	0.571	0.573	0.146	−0.151	0.810
39–49	0.657	0.623	−0.139	−0.110	0.971
50–61	0.700	0.622	0.016	−0.558	0.443
61–72	0.613	0.540	−0.221	−0.200	0.700
CHAIN	0.661	0.607	−0.012	0.089	0.772
**Chain F—Protofibril**					
**Fragment**	**RD**		**Correlation Coefficient**		
	**T-O-R**	**T-O-H**	**HvT**	**TvO**	**HvO**
1–6	0.668	0.553	−0.005	0.064	0.875
16–26	0.615	0.558	0.286	−0.144	0.875
23–30	0.572	0.573	0.143	−0.155	0.809
39–49	0.657	0.623	−0.139	−0.110	0.971
50–61	0.700	0.621	0.016	−0.558	0.442
61–72	0.612	0.539	−0.221	−0.200	0.698
CHAIN	0.661	0.607	−0.012	0.089	0.772
**Chain E—Individual**					
**Fragment**	**RD**		**Correlation Coefficient**		
	**T-O-R**	**T-O-H**	**HvT**	**TvO**	**HvO**
1–6	0.741	0.372	−0.093	−0.037	0.543
16–26	0.676	0.433	0.286	−0.387	0.696
23–30	0.582	0.387	0.117	−0.228	0.620
39–49	0.687	0.388	−0.114	0.017	0.888
50–61	0.700	0.489	0.057	−0.537	0.116
61–72	0.656	0.398	−0.237	−0.240	0.507
CHAIN	0.679	0.430	−0.027	0.095	0.548
**Chain F—Individual**					
**Fragment**	**RD**		**Correlation Coefficient**		
	**T-O-R**	**T-O-H**	**HvT**	**TvO**	**HvO**
1–6	0.741	0.372	−0.093	−0.037	0.543
16–26	0.676	0.433	0.286	−0.387	0.696
23–30	0.582	0.387	0.117	−0.228	0.620
39–49	0.687	0.388	−0.114	0.017	0.888
50–61	0.700	0.489	0.057	−0.537	0.115
61–72	0.656	0.397	−0.233	−0.240	0.507
CHAIN	0.679	0.430	−0.027	0.095	0.548

**Table 5:** RD values obtained using I-Tasser, Robetta and FOD. In addition to RD values (for T-O-R and T-O-H) the table also lists HvT, TvO and HvO correlation coefficients. The division into fragments is consistent with the one shown in Figure 4. Values in bold distinguish the status accordant with FOD model (RD < 0.5).

**I-Tasser**					
**IT-1**	**RD**		**Correlation Coefficient**		
**Fragment**	**T-O-R**	**T-O-H**	**HvT**	**TvO**	**HvO**
1–6	0.411	0.142	0.032	0.600	0.469
16–26	0.509	0.200	0.308	0.659	0.706
23–30	0.641	0.357	0.308	0.219	0.415
39–49	0.494	0.283	0.501	0.306	0.841
50–61	0.614	0.618	0.169	0.196	0.466
61–72	0.627	0.232	0.089	0.176	0.559
CHAIN	0.519	0.266	0.200	0.473	0.574
**IT-2**	**RD**		**Correlation Coefficient**		
**Fragment**	**T-O-R**	**T-O-H**	**HvT**	**TvO**	**HvO**
1–6	0.825	0.138	−0.016	0.551	0.242
16–26	0.607	0.279	0.372	0.039	0.730
23–30	0.503	0.236	0.336	0.226	0.651
39–49	0.765	0.313	−0.230	−0.358	0.849
50–61	0.667	0.548	0.027	−0.416	0.122
61–72	0.605	0.367	−0.486	−0.133	0.475
CHAIN	0.635	0.353	−0.028	0.172	0.519
**IT-3**	**RD**		**Correlation Coefficient**		
**Fragment**	**T-O-R**	**T-O-H**	**HvT**	**TvO**	**HvO**
1–6	0.729	0.258	−0.438	−0.109	0.416
16–26	0.558	0.179	0.381	0.567	0.830
23–30	0.408	0.173	0.607	0.635	0.805
39–49	0.369	0.191	0.282	0.659	0.828
50–61	0.701	0.702	0.102	−0.196	0.587
61–72	0.545	0.285	0.212	0.162	0.638
**CHAIN**	**0.478**	0.262	0.270	0.524	0.684
**IT-4**	**RD**		**Correlation Coefficient**		
**Fragment**	**T-O-R**	**T-O-H**	**HvT**	**TvO**	**HvO**
1–6	0.407	0.183	0.216	0.702	0.554
16–26	0.817	0.230	0.191	0.125	0.730
23–30	0.746	0.468	0.253	−0.453	0.345
39–49	0.432	0.140	0.190	0.494	0.818
50–61	0.630	0.585	0.426	0.094	0.367
61–72	0.601	0.350	0.412	0.193	0.781
CHAIN	0.605	0.315	0.229	0.252	0.581
**IT-5**	**RD**		**Correlation Coefficient**		
**Fragment**	**T-O-R**	**T-O-H**	**HvT**	**TvO**	**HvO**
1–6	0.545	0.138	−0.024	0.198	0.590
16–26	0.537	0.232	0.321	0.313	0.840
23–30	0.663	0.323	0.254	0.040	0.670
39–49	0.504	0.162	0.444	0.426	0.848
50–61	0.723	0.862	0.194	0.208	0.872
61–72	0.477	0.257	0.501	0.524	0.858
CHAIN	0.513	0.327	0.260	0.418	0.659
**Robetta**					
**ROB-1**	**RD**		**Correlation Coefficient**		
**Fragment**	**T-O-R**	**T-O-H**	**HvT**	**TvO**	**HvO**
1–6	0.627	0.474	0.010	0.165	0.633
16–26	0.473	0.380	0.687	0.400	0.808
23–30	0.566	0.619	0.619	0.157	0.830
39–49	0.606	0.381	0.042	0.275	0.889
50–61	0.650	0.495	0.070	−0.105	0.376
61–72	0.675	0.405	−0.27	−0.182	0.594
CHAIN	0.745	0.568	−0.001	0.109	0.683
**ROB-2**	**RD**		**Correlation Coefficient**		
**Fragment**	**T-O-R**	**T-O-H**	**HvT**	**TvO**	**HvO**
1–6	0.535	0.345	−0.300	0.218	0.690
16–26	0.601	0.518	0.448	0.004	0.809
23–30	0.621	0.675	0.388	−0.147	0.824
39–49	0.839	0.292	0.014	0.036	0.904
50–61	0.681	0.531	0.083	−0.297	0.404
61–72	0.699	0.364	−0.250	0.017	0.552
CHAIN	0.656	0.422	0.032	0.249	0.611
**ROB-3**	**RD**		**Correlation Coefficient**		
**Fragment**	**T-O-R**	**T-O-H**	**HvT**	**TvO**	**HvO**
1–6	0.600	0.382	−0.495	−0.012	0.623
16–26	0.523	0.410	0.576	0.144	0.795
23–30	0.572	0.510	0.463	−0.038	0.821
39–49	0.842	0.473	−0.173	−0.189	0.955
50–61	0.646	0.512	0.127	−0.168	0.394
61–72	0.698	0.396	−0.182	0.226	0.576
CHAIN	0.682	0.444	0.074	0.351	0.650
**ROB-4**	**RD**		**Correlation Coefficient**		
**Fragment**	**T-O-R**	**T-O-H**	**HvT**	**TvO**	**HvO**
1–6	0.624	0.519	−0.013	0.141	0.766
16–26	0.441	0.329	0.714	0.464	0.806
23–30	0.593	0.557	0.618	0.171	0.831
39–49	0.782	0.453	−0.120	−0.072	0.939
50–61	0.658	0.575	0.160	−0.187	0.344
61–72	0.678	0.346	−0.187	0.016	0.568
CHAIN	0.748	0.542	0.022	0.154	0.657
**ROB-5**	**RD**		**Correlation Coefficient**		
**Fragment**	**T-O-R**	**T-O-H**	**HvT**	**TvO**	**HvO**
1–6	0.545	0.111	−0.045	0.571	0.558
16–26	0.701	0.341	0.201	0.166	0.916
23–30	0.748	0.314	0.251	−0.284	0.666
39–49	0.372	0.220	0.255	0.676	0.809
50–61	0.643	0.712	0.258	0.398	0.640
61–72	0.432	0.155	0.513	0.587	0.794
CHAIN	0.566	0.351	0.272	0.375	0.733
**FOD**					
**FOD-1**	**RD**		**Correlation Coefficient**		
**Fragment**	**T-O-R**	**T-O-H**	**HvT**	**TvO**	**HvO**
1–6	0.774	0.288	−0.555	−0.040	0.023
16–26	0.346	0.116	0.238	0.643	0.503
23–30	0.808	0.041	0.510	−0.319	0.339
39–49	0.602	0.187	−0.264	0.267	0.606
50–61	0.733	0.714	0.369	−0.291	0.457
61–72	0.568	0.217	−0.269	0.279	0.200
CHAIN	0.503	0.253	0.126	0.563	0.390
**FOD-2**	**RD**		**Correlation Coefficient**		
**Fragment**	**T-O-R**	**T-O-H**	**HvT**	**TvO**	**HvO**
1–6	0.502	0.017	−0.687	0.723	−0.013
16–26	0.315	0.110	0.165	0.770	0.527
23–30	0.675	0.261	0.249	0.105	0.521
39–49	0.543	0.271	−0.175	0.258	0.693
50–61	0.593	0.698	0.375	0.092	0.706
61–72	0.522	0.191	−0.211	0.268	0.268
**CHAIN**	**0.364**	0.191	0.093	0.665	0.410
**FOD-3**	**RD**		**Correlation Coefficient**		
**Fragment**	**T-O-R**	**T-O-H**	**HvT**	**TvO**	**HvO**
1–6	0.605	0.104	−0.373	0.440	0.386
16–26	0.325	0.107	0.054	0.700	0.552
23–30	0.580	0.194	0.328	0.127	0.574
39–49	0.575	0.187	−0.171	0.313	0.689
50–61	0.676	0.682	0.331	−0.282	0.554
61–72	0.338	0.080	0.135	0.603	0.395
**CHAIN**	**0.369**	0.175	0.152	0.635	0.414
**FOD-4**	**RD**		**Correlation Coefficient**		
**Fragment**	**T-O-R**	**T-O-H**	**HvT**	**TvO**	**HvO**
1–6	0.492	0.137	−0.349	0.443	0.231
16–26	0.387	0.136	0.224	0.601	0.546
23–30	0.705	0.470	0.301	−0.414	0.290
39–49	0.589	0.140	−0.211	0.453	0.410
50–61	0.840	0.744	−0.151	−0.366	0.252
61–72	0.556	0.319	0.038	0.092	0.357
CHAIN	0.648	0.346	0.059	0.150	0.395
**FOD-5**	**RD**		**Correlation Coefficient**		
**Fragment**	**T-O-R**	**T-O-H**	**HvT**	**TvO**	**HvO**
1–6	0.620	0.075	−0.363	0.484	0.004
16–26	0.513	0.121	0.025	0.576	0.446
23–30	0.668	0.247	−0.113	0.031	0.070
39–49	0.806	0.205	−0.18	0.220	0.646
50–61	0.807	0.588	−0.458	−0.326	0.033
61–72	0.638	0.344	−0.154	−0.022	0.348
CHAIN	0.644	0.313	−0.049	0.200	0.357

**Table 6:** Parameters describing the 541–553 fragment (chain A) of the tau protein Tpp in complex with the ww domain (residues 6–44).

Tpp	RD		Correlation Coefficient		
	T-O-R	T-O-H	HvT	TvO	HvO
Complex	0.680	0.546	0.171	0.235	0.700
Chain A (tau) in complex	0.452	0.152	0.365	0.730	0.756
Chain A(tau) individual	0.630	0.240	0.080	0.348	0.753

**Table 7:** Properties of peptides which match the N-terminal fragment of the tau protein. The table lists PDB ID, sequences (indicating which fragments are identical to the tau chain) and RD (T-O-R) values. Positions + aa inform about positions of amino acids present in the chain but these residues are not the object of analysis.

Peptide	Sequence	Position	RD
Tau (306–311)	VQIVYK	306–311	0.164
Tau (306–310)	VQIVYK + LA	306–311 + 2 aa	0.332
Tau (306–311B)	VQIVYK	306–311	0.160
Tau (305–311)	AS + VQIVYK + AEFYK	2 aa + 306–311 + 5 aa	0.707
Tau (623–628)	VQIVYK	306–311	0.174
Tau (306–311C)	VQIVYK	306–311	0.170

**Table 8:** Parameters describing the microfilament structure as present in F-actin. β-sheet-1—sheet with starting numbers 8–11, β-sheet-2—sheet with starting numbers 150–154, β-sheet-3—sheet with starting numbers 70–72. Stop sign—fragment “stopping” linear propagation: *—fragment 351–374, **—fragment 325–331, ***—fragment 173–176, No neg CC—status of the chain with residues representing negative correlation coefficients (CC) for HvT and TvO with high HvO, No selected—status of the chain with residues identified as discordant under visual analysis of the T and O profile—shown in Figure 9 and Figure 10.

F-actin	RD		Correlation Coefficient		
	T-O-R	T-O-H	HvT	TvO	HvO
Complex	0.783	0.704	0.058	0.081	0.722
Chain F in complex	0.614	0.512	0.111	0.155	0.730
β-sheet-1 *	0.743	0.465	−0.163	−0.411	0.723
β-sheet-2 **	0.593	0.415	−0.08	−0.036	0.635
β-sheet-3 ***	0.532	0.593	−0.398	−0.253	0.939
Stop sign β1	0.445	0.346	0.504	0.420	0.867
No neg CC	0.605	0.467	0.121	0.179	0.717
No selected	0.488	0.391	0.312	0.425	0.748
C-term	0.445	0.346	0.504	0.420	0.867
Chain F individual	0.641	0.544	0.140	0.382	0.709
P-P	0.754	0.594	0.062	0.136	0.687
No P-P	0.635	0.543	0.158	0.357	0.737
β-sheet-1 *	0.774	0.503	−0.161	−0.393	0.721
β-sheet-2 **	0.556	0.402	−0.137	−0.367	0.489
β-sheet-3 ***	0.438	0.507	0.221	0.460	0.940
Stop sign β1	0.492	0.376	0.610	0.414	0.866
No neg CC	0.615	0.502	0.209	0.402	0.714
No selected	0.487	0.400	0.338	0.528	0.729
C-term	0.492	0.376	0.610	0.414	0.866

**Table 9:** Set of proteins subjected to analysis, along with an indication of chain length and complexation capabilities. The rightmost column provides references.

PDB ID	Characteristics	Length	Complex	Reference
Tau—amyloid				
5O3O phf-tauO	Microtubule-associated protein tau	73 aa	10 chains	[11]
5O3L phf-tauL	Microtubule-associated protein tau	73 aa	10 chains	[11]
5O3T phf-tauT	Microtubule-associated protein tau	73 aa	10 chains	[11]
Tau—non-amyloid				
2MZ7 Tau (267–312)	Tau (267–312) bound to microtubules	46 aa		[34]
1I8H Tpp	Pin1 ww domain complexed with human tau phosphothreonine peptide	14 aa	Complex	[33]
Peptides				
2ON9 Tau (306–311A)	Amyloid forming peptide VQIVYK from the repeat region of tau (in tau 306-311)	6 aa		[47]
3Q9G Tau (306–310)	VQIVY segment from Alzheimer’s tau displayed on 42-membered macrocycle scaffold Cyclic pseudo-peptide vqiv(4bf)(orn)(hao)kl(orn)	5 aa		[48]
3OVL Tau (306–311B)	Microtubule-associated protein. VQIVYK (residues 306–311)	6 aa		[49]
4E0M Tau (305–311)	SVQIVYK segment from human tau (305–311) displayed on 54-membered macrocycle scaffold (form i)	7 aa		[50]
4NP8 Tau (623–628)	Structure of an amyloid forming peptide VQIVYK from the second repeat region of tau (alternate polymorph) (in tau 623–628)	7 aa		[51]
5K7N Tau (306–311C)	tau VQIVYK peptide	6 aa		[52]
Fibrilar form as appears in microfilament				
F-actin	Actin, alpha skeletal muscle	375 aa		[53]
